# Improving efficiency in the radiation management of multiple brain metastases using a knowledge‐based planning solution for single‐isocentre volumetric modulated arc therapy (VMAT) technique

**DOI:** 10.1002/jmrs.526

**Published:** 2021-07-26

**Authors:** James O’Toole, Maddison Picton, Mario Perez, Michael Back, Dasantha Jayamanne, Andrew Le, Kenny Wu, Chris Brown, John Atyeo

**Affiliations:** ^1^ Northern Sydney Cancer Centre Royal North Shore Hospital Sydney New South Wales Australia; ^2^ Genesis Care Sydney New South Wales Australia; ^3^ The Brain Cancer Group North Shore Private Hospital Sydney New South Wales Australia; ^4^ Sydney Medical School University of Sydney Sydney New South Wales Australia; ^5^ National Health and Medical Research Council Clinical Trials Centre University of Sydney Sydney New South Wales Australia

**Keywords:** volumetric modulated arc therapy (VMAT), neurological, radiotherapy (radiation therapy), radiation oncology, stereotactic radiosurgery (SRS)

## Abstract

**Introduction:**

This study aimed to develop a single‐isocentre volumetric modulated arc therapy (si‐VMAT) technique for multiple brain metastases using knowledge‐based planning software, comparing it with a multiple‐isocentre stereotactic radiosurgery (mi‐SRS) planning approach.

**Methods:**

Twenty‐six si‐VMAT plans were created and uploaded into RapidPlan^TM^ (RP) to create a si‐VMAT model. Ten patients, with 2 to 6 metastases (mets), were planned with a si‐VMAT technique utilising RP, and a mi‐SRS technique on Brainlab iPlan.

Paddick Conformity Index (PCI) was used to compare conformity. The volumes of the brain receiving 15Gy, 12Gy, 10Gy, 7.5Gy and 3Gy were also compared.

Retrospective treatment times from the last eight patients treated were averaged for pre‐imaging and beam on time to calculate treatment times for both techniques.

**Results:**

There was a significant difference in the PCI scores for the mi‐SRS plans (*M* = 0.667, SD = 0.114) and si‐VMAT plans (*M* = 0.728, SD = 0.088), with PCI values suggesting better prescription dose conformity with the si‐VMAT technique (*P* = 0.014). Percentage of total brain volume receiving low‐dose wash at four of the five different dose levels was significantly less (*P* < 0.05) with mi‐SRS.

Average time to treat a single met with current mi‐SRS technique is 25.7 min, with each additional met requiring this same amount of time. The average time to treat 2–3 mets using si‐VMAT would be 25.3 min and 4+ metastases 33.5 min.

**Conclusion:**

A knowledge‐based si‐VMAT approach was efficient in planning and treating multi metastases while achieving clinically acceptable dosimetry with respect to dose conformity and low‐dose fall off.

## Graphical abstract

This study aimed to develop a single‐isocentre volumetric modulated arc therapy (si‐VMAT) technique for multiple brain metastases (MBM) using knowledge‐based planning software, comparing it with a multiple isocentre stereotactic radiosurgery (mi‐SRS) planning approach. A knowledge based si‐VMAT approach was efficient in planning and treating multi metastases while achieving clinically acceptable dosimetry with respect to dose conformity and low‐dose fall off.

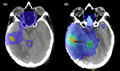

## Introduction

Increasing clinical trial evidence in the management of brain metastases (BM) from solid tumours has demonstrated that focal radiation therapy for oligometastases (usually defined as 1–4 lesions) results in equivalent survival and less late neurocognitive morbidity compared with whole brain radiation therapy.^1^, ^2^, ^3^, ^4^, ^5^, ^6^, ^7^ There may also be an emerging role for focal radiotherapy in carefully selected patients with multiple low‐volume BM (≥5 lesions).^8^, ^9^

Stereotactic radiosurgery (SRS) is a well‐established modality for the focal treatment of multiple intracranial metastases. The technique delivers highly conformal doses to each individual target volume through individually planned multiple‐beam techniques. However, with increasing utilisation and numbers of lesions treated, there are significant resources required for planning, as well as subsequent longer treatment duration impacting on machine workflow and patient comfort and tolerability.^10^

With these limitations in mind, there has been a push to find less resource intensive solutions with the newer technologies that are available. A single‐isocentre volumetric modulated arc therapy (si‐VMAT) technique has the potential to treat multiple brain metastases more efficiently and deliver equivalent dosimetric endpoints compared to SRS.^11^, ^12^, ^13^, ^14^, ^15^ Knowledge‐based planning techniques have the ability to further improve the efficiency of si‐VMAT planning.^16^

This study aimed to evaluate the quality and efficiency of standard multiple‐isocentre SRS (mi‐SRS) planning for multiple BMs created on Brainlab’s iPlan planning software, to a si‐VMAT technique planned with a department developed si‐VMAT RapidPlan^TM^ (RP) (v 13.6.23) model on the Eclipse planning system (v13.6.23)(Varian Medical Systems, Palo Alto, USA).

## Methods

### Patient selection

Consecutive adult patients diagnosed with BM and referred to our Department of Radiation Oncology were entered into a prospective database approved by the Northern Sydney Local Health District Ethics Review Board. Eligible patients for this dosimetry study were selected from the database and had previously been treated for multiple BM. Due to the limited number of patients and no previous use of a si‐VMAT technique in the department, all available data had to be used to create and validate a si‐VMAT RapidPlan model. There were 35 patient data sets treated at our centre that had multiple metastases (mets) contoured either over one course or many courses of treatment. Nine patients with six or less mets were chosen at random to set aside as validation patients for the model. One Trans‐Tasman Radiation Oncology Group computed tomography (CT) and contour data set that had five mets was also used as a tenth validation patient. These validation patients could not be used in the creation of the model. The remaining 26 patients were replanned manually using the proposed si‐VMAT technique to create the model.

### RapidPlan model creation

An RP model requires the creation of a library from the plans of previously treated patients, with a minimum number of 20 patients to create a model. The selected patients’ original radiation therapy planning data, including planning CT, fused diagnostic MRI and contoured brain mets, were exported from the Eclipse planning system (v13.6.23) into the RP model configuration to train the model. Using this data, mathematical parameters were generated through the analysis of geometric and dosimetric statistics of the 26 uploaded patient’s plans and contours, with selected patients replanned with a si‐VMAT technique.

Planning target volume (PTV) structures prescribed to the same dose were combined into one structure called PTV high dose (PTV HD) and exported to the model to work around the known issues that v13.6.23 of the RP software has modelling small structures. If there was more than one dose, structures would be combined into one structure in their respective dose levels and called PTV intermediate dose (PTV ID) and PTV low dose (PTV LD) and exported to the model.

### Model verification

RP allows the verification and comparison of dose and contour statistics within model configuration. Statistics such as volume size, overlap with target, geometric distribution and predicted DVH can be used to alert the user of potential outliers. Because of the small number of patients left to use in the model, a more lenient approach was taken to the outlier structures and their inclusion in the model.

The predicted DVH tool and the bandwidth it provides for where the estimated DVH for an organ at risk (OAR) should fall was also used to identify plan quality outliers. The estimated DVH tool used other plans contours and DVH statistics within the model to create the estimations. If a plan was deemed to be of poor quality, it would be replanned to a better standard and then replaced within the model. All plans were assessed as being of good quality, with no replanning required.

### Planning technique

#### Single‐isocentre VMAT technique

A VMAT beam arrangement was used to plan these cases. The isocentre was placed in the geographic centre of all the BM being treated. Collimator angles were chosen to optimise the multi‐leaf collimator (MLC) position, allowing the most time to modulate between metastases for each arc. The plans are dosed to 100% with the model designed to have maximums in each PTV around 125% of the prescription dose. A summary of the number of beams and couch angles used depending on the number of brain metastases and their location are presented in Table 1. The plans were calculated using Eclipse version 13.6.23. In the optimising window the grid size was set to the smallest setting of 1.25mm. The optimiser was paused at step 4/5 in the multi‐resolution (MR) level 1 for all plans until the objective function chart flattened for 2–3 min. The intermediate dose option was not used and the final calculation was done using calculation model AAA_13623 on 1mm dose grid.

**Table 1:** Beam arrangement for number of brain metastases (BM) and their laterality.

Number of BM	Laterality	Technique
2–3	Right	4 Arcs 2 arcs, 360 degrees, couch 0 1 arc, 180 degrees, couch 315 1 arc, 180 degrees, couch 270
2–3	Left	4 Arcs 2 arcs, 360 degrees, couch 0 1 arc, 180 degrees, couch 45 1 arc, 180 degrees, couch 90
4+	NA	5 Arcs 2 arcs, 360 degrees, couch 0 1 arc, 180 degrees, couch 315 1 arc, 180 degrees, couch 90 1 arc, 180 degrees, couch 45

#### Multiple‐isocentre SRS Technique

Patients planned using the iPlan (v4.5.3) planning system had an isocentre placed in the centre of each PTV and were dosed to the 80% line with the maximum at 100%. Cones were used to plan for PTVs under 1.5 cm and MLC dynamic arcs were available to use to plan PTV’s larger than 1.5cm as per our protocol. There were no limitations in arc number or couch angle for each PTV but the starting template initially defaulted to three arcs and three couch angles. The calculation algorithm used in iPlan was Pencil Beam Algorithm.

### Comparison method

The ten validation patients not included in the RP model were planned with both techniques for comparison. Each patient had between 2 and 6 metastases and was prescribed 20 Gy in a single fraction to at least 99% of the volume. Total brain volume and total brain met volume in the 10 validation patients also varied as seen in Table 3.

As an objective comparison between two treatment plans, the conformity index equation proposed by Paddick^17^ was used to assess the conformity of the prescription dose to the target volumes. The Paddick conformity index (PCI) formula is commonly used by other authors and will not give false scores.

$$\text{PCI}={\text{TV}}_{\text{PIV}}^{2}/\left(\text{TV}\times V_{\text{RI}}\right).$$

TV_PIV_ = Target volume covered by the prescription isodose.

V_RI_ = Total volume covered by the prescription isodose.

The primary dosimetric endpoint for the study was the difference in the average PCI between the RP si‐VMAT plans and the mi‐SRS plans.

The volume of brain treated with 15 Gy, 12 Gy, 10 Gy, 7.5 Gy and 3 Gy was also assessed using DVH table results to determine the low‐dose drop off difference between the two techniques.

### Timing calculations

To determine the length of time to treat both techniques, the treatment times for the last eight SRS patients were assessed to determine the average beam on time for a single arc and also the average time it took for pre beam imaging and couch adjustments. Beam on time for si‐VMAT plans was measured by delivering the fluences in QA mode on the machine and average time was calculated. These averages would then be applied to both techniques to approximate the treatment times for the number of metastases treated.

## Results

### RapidPlan model

Twenty‐six plans were used to create the model. No plans or contours were removed when analysing the outlier statistics within RP Model Configuration. Six OAR contours were used in the make‐up and training of the model. Table 2 lists the OAR contours in the model and the number of OAR contours that make up the model.

**Table 2:** Number of organ at risk (OAR) contours in the RapidPlan (RP) model.

OAR Contour	Number in RP model
Brain	26
Brain Stem	25
Eyes	32
Lens	47
Optic Chiasm	12
Optic Nerve	32

### PCI comparison

Data were first tested for normality using the Shapiro–Wilk test, with testing of the null hypothesis of no difference indicating normal distribution of PCI values for both the mi‐SRS plans (*P* = 0.072) and si‐VMAT plans (*P* = 0.835). A paired‐samples *t*‐test was conducted to compare the difference in the PCI for the mi‐SRS and si‐VMAT plans. A comparison of the PCI between the mi‐SRS and si‐VMAT plans are presented in Table 3. There was a significant difference in the scores for the mi‐SRS plans (*M* = 0.667, SD = 0.114) and si‐VMAT plans (*M* = 0.728, SD = 0.088); *t*(9) = 3.063, *P* = 0.014, indicating that the si‐VMAT technique had better conformity of the prescription dose compared to the mi‐SRS plans.

**Table 3:** Patient demographic features and comparison of Paddick conformity index (PCI) results between multiple‐isocentre stereotactic radiosurgery (mi‐SRS) using iPlan and single‐isocentre volumetric modulated arc therapy (si‐VMAT) using RapidPlan.

Patient	No. mets	Total brain volume(cc)	Total brain met volume(cc)	PCI si‐VMAT	PCI mi‐SRS	Difference in PCI (si‐VMAT – mi‐SRS)
1	5	1297	1.89	0.65	0.66	−0.01
2	5	1522	5.84	0.78	0.64	0.14
3	4	1269	3.28	0.76	0.72	0.04
4	6	1366	5.13	0.77	0.75	0.02
5	4	1427	4.56	0.68	0.68	0.00
6	3	1340	5.20	0.85	0.79	0.06
7	6	1277	10.62	0.82	0.73	0.09
8	2	1108	0.90	0.69	0.56	0.13
9	2	1364	0.85	0.73	0.74	−0.01
10	5	1200	1.67	0.55	0.40	0.15
Mean	‐	1317	4.01	0.73	0.67	0.06
95% CI	‐	‐	‐	‐	‐	[0.01, 0.11]

mets = metastases; cc = cubic centimetres.

### Brain volume comparison

**Figure 1:**
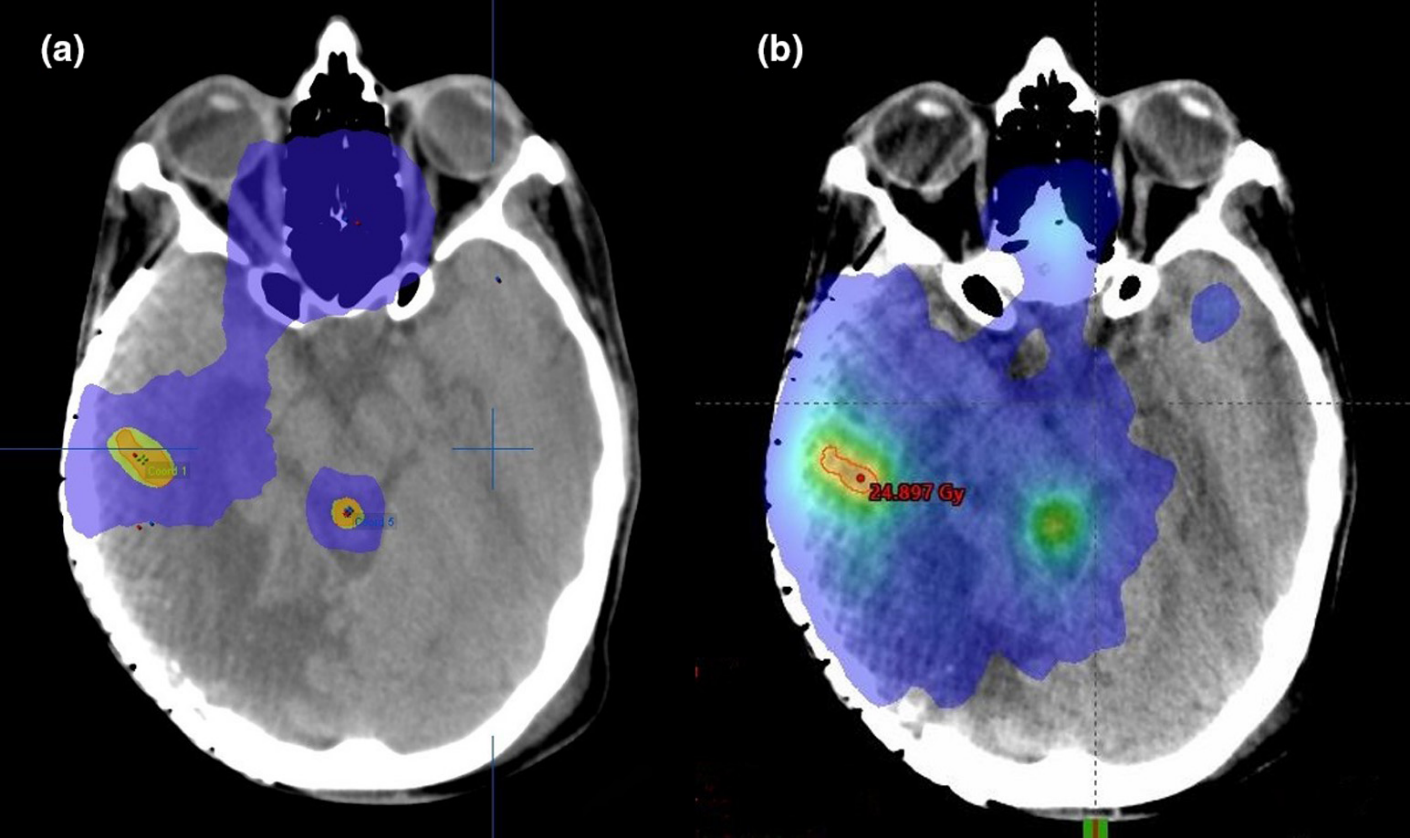
3 Gy dose colour wash comparison (in blue) between (a) the mi‐SRS Plan and (b) si‐VMAT plan.

**Figure 2:**
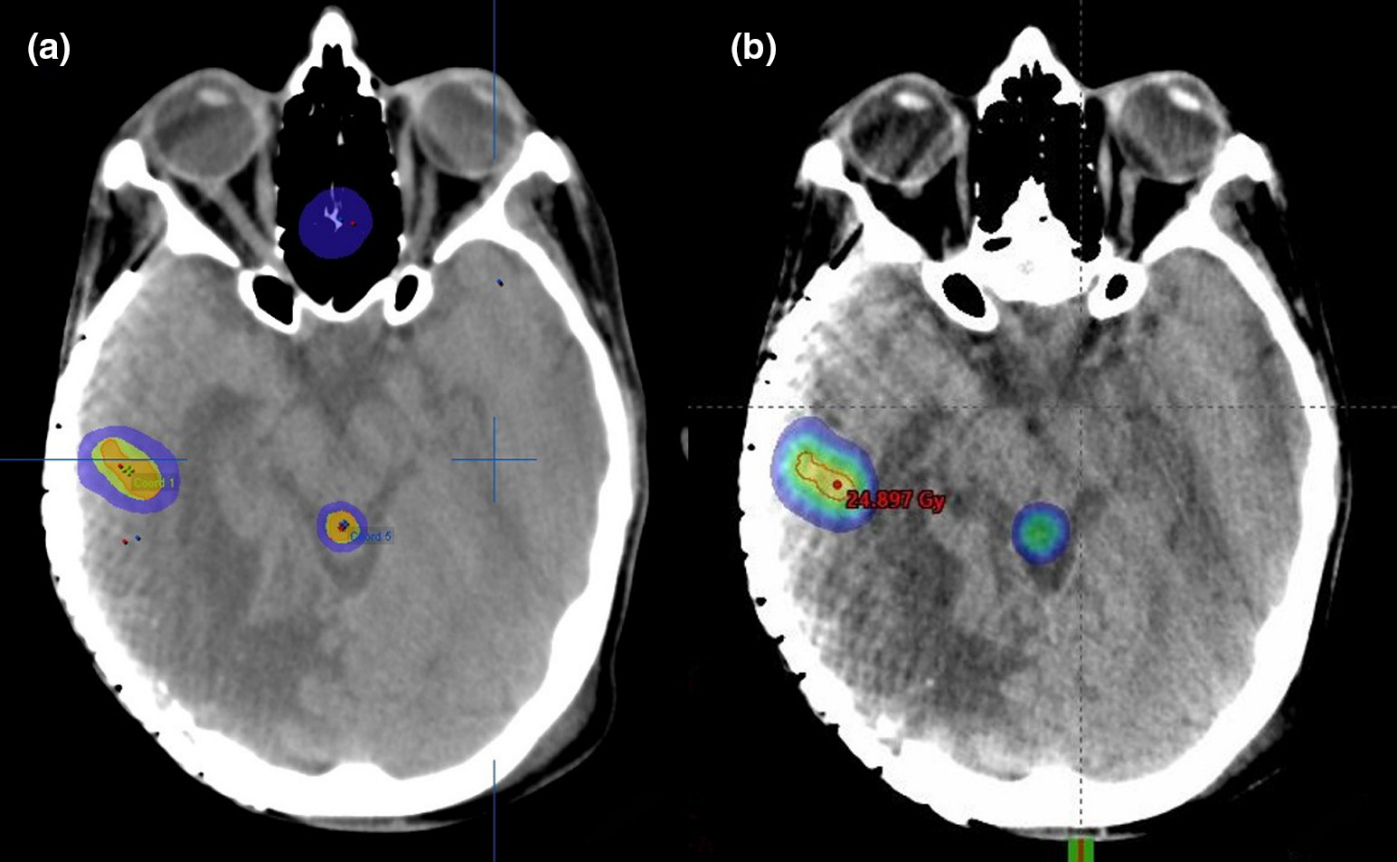
10 Gy dose colour wash comparison between (a) the mi‐SRS Plan and (b) si‐VMAT plan.

Comparisons of the two planning techniques in relation to the percentage of total brain volume receiving dose wash at five different dose levels (15 Gy, 12 Gy, 10 Gy, 7.5 Gy, 3 Gy) were also conducted using the Wilcoxon signed rank test. Results indicated that the mi‐SRS technique planned using iPlan, was superior in reducing the volume of brain receiving dose at all dose levels (Table 4). Apart from the 12 Gy dose level, volumes treated at the other four dose levels were significantly less using the mi‐SRS technique at the *P* < 0.05 level. In a patient with five metastases, Figure 1 shows the dosimetric difference in the 3 Gy low‐dose colour wash between the mi‐SRS and si‐VMAT plan in axial view. Figure 2 shows the difference in the 10 Gy colour wash between the two techniques for the same patient.

**Table 4:** Comparison of mean brain volumes receiving 15 Gy, 12 Gy, 10 Gy, 7.5 Gy or 3 Gy between multi isocentre stereotactic radiosurgery (mi‐SRS) and single‐isocentre volumetric modulated arc therapy (si‐VMAT) plans.

Variable	Mean (SD) *n* = 10		Median difference (95% CI)	*P‐*value^a^
	mi‐SRS	si‐VMAT		
15 Gy Brain volume (cc)	8.62 (5.89)	9.86 (6.01)	1.27 (0.00, 2.28)	0.028
12 Gy Brain volume (cc)	12.98 (8.16)	15.18 (8.74)	2.42 (−1.28, 4.92)	0.059
10 Gy Brain volume (cc)	15.75 (10.42)	21.15 (11.99)	4.59 (3.17, 8.21)	0.005
7.5 Gy Brain volume (cc)	24.31 (16.66)	34.73 (20.43)	9.36 (6.18, 16.38)	0.005
3 Gy Brain volume (cc)	128.54 (108.51)	240.06 (178.76)	105.49 (55.27, 170.16)	0.005

SD, standard deviation; CI, confidence interval; Gy, Gray; cc, cubic centimetres.

^a^ Wilcoxon signed‐rank test.

The average measured beam on time for a mi‐SRS arc was 62 sec compared to 39 sec measured for si‐VMAT. Average time that was calculated pre imaging and bed movements before the beam delivery was started was 7 min and 32 sec. Table 5 shows the time comparisons of the calculated treatment time between mi‐SRS and si‐VMAT.

**Table 5:** Comparison of the averaged treatment times between multi isocentre stereotactic radiosurgery (mi‐SRS) and single‐isocentre volumetric modulated arc therapy (si‐VMAT) for the different number of metastases treated.

Metastases treated	mi‐SRS treatment time	si‐VMAT treatment time
2	51 mins 24 sec	25 mins 20 sec
4	102 mins 48 sec	33 mins 31 sec
6	154 mins 12 sec	33 mins 31 sec
8	205 mins 36 sec	33 mins 31 sec
10	257 mins	33 mins 31 sec

## Discussion

This dosimetric study demonstrated that a si‐VMAT technique produced an increased conformity of the prescription dose to the PTV compared to our current mi‐SRS technique using cones or dynamic arcs. Additionally a potentially large improvement in utilisation of departmental planning and treatment resources was demonstrated with significant time savings in both areas increasing with the number of metastases being treated.

As each BM requires its own plan and extended treatment duration, using mi‐SRS can place stress on a department’s resources. With these patients having advanced metastatic disease and often being potentially unwell, the long treatment times can require multiple different appointment sessions over many days to give patients a break and improve their compliance in completing their treatment.

The dosimetric improvements were in agreement with the results by Hardcastle et al,^10^ who compared plan quality of a si‐VMAT technique against a standard conformal arc technique and reported better conformity of the prescription dose to the targets with the si‐VMAT technique. We also found better conformity of the prescription dose to the targets with the si‐VMAT technique compared to the mi‐SRS plans where cones are mainly used. With the assistance of 2.5mm thickness MLC leaves and the optimising algorithm, it is not surprising that the conformity of the prescription is better for PTV structures that are irregular in shape.

Similar to Thomas et al.,^11^ we found that we could achieve clinically acceptable dose drop off to the brain as far down as 7.5 Gy, which is the 30% line used in our centre, to visually determine if there is fast drop off in mi‐SRS plans. Although the volume of brain treated using the si‐VMAT technique was on average greater than the volume treated using the mi‐SRS technique, the volume differences were small (Table 4). The median difference in the volume of brain treated with 15 Gy and 12 Gy was respectively 1.27cc and 2.42cc more using the si‐VMAT technique for the 10 validation patients. For 10 Gy and 7.5 Gy the median difference in the volume of brain treated was 4.59cc and 9.36cc more using the si‐VMAT technique, which equates to 0.4 and 0.9% more volume of brain. At the very low doses of the prescription, the mi‐SRS technique holds an advantage. Leaf leakage comes into play when using a si‐VMAT technique, and a median difference of 105.5cc more of the brain received 3 Gy, which equates to 8.8%. Whilst statistically significant differences were noted favouring the mi‐SRS technique, no studies have yet correlated toxicity with this low‐dose wash. At doses where a potential clinical impact may be recognised (i.e. at 12 Gy and 15 Gy brain dose levels) the median volumetric differences between the two techniques were in each case less than 2.5cc.

Further improvements may be achieved through implementation of jaw tracking. This feature, which brings the jaw in to match the most outward PTV, would reduce some leakage and decrease the amount of low dose to the brain further. How much improvement in low dose will be tested once the feature is commissioned, but it is not expected to match the low dose numbers of the mi‐SRS technique.

Future versions of RP will also allow smaller contours to be used in the estimation process. Currently each PTV is combined together and expanded by 1mm to create one planning structure. This has limitations as the optimiser sees only one structure and ensures TD covers 99% of this volume resulting in some PTV’s being over covered. The ability to have smaller structures will allow each PTV to be attached and optimised individually which should result in a more conformal plan with better PCI results.

A si‐VMAT technique has efficiency benefits for both planning and treatment. Regardless of the number of metastases the si‐VMAT plan will take on average 45 min to plan and 40 min to treat. By comparison, mi‐SRS plans need a plan per metastasis. At our centre, each BM would take approximately 45 min to plan and 20 min to treat. The more metastases that need to be treated the more time will be saved in planning and on the treatment machine using the si‐VMAT technique. It should be noted that planning was done for delivery of treatment on a Varian Medical Systems TrueBeam^TM^STx system. Operation of the treatment unit in flattening filter‐free (FFF) mode and energy used (6X versus 10X) will have an impact on the overall time.

A RP solution for these si‐VMAT plans allows planning staff with little experience planning mi‐SRS treatments an opportunity to plan these cases, as it simplifies the process. This eases the burden of having to continually train staff with the skills to plan SRS treatments on the iPlan planning system or having trained staff rostered in planning at all times to be available to plan these cases as they come through the clinic.

Being capable of managing these patients with multiple brain metastases using good quality plans that utilise current linear accelerators that treat other body sites is a more cost‐effective approach for resource allocation within a department and across a large healthcare system. The alternative is having dedicated SRS treatment machines that can be expensive such as Cyberknife and Gamma Knife. Further developments in si‐VMAT techniques will allow more access for patients presenting with multiple brain metastases to have SRS as a treatment option, as more centres will have the capacity to invest in this direction rather than in dedicated SRS treatment units.

It must be noted that a small limitation of this study is that the DVH values for each technique were sourced from tables specific to each planning system. It is likely that because of this, a less accurate comparison resulted than if the DVH from one planning system was used to compare values. This difference, however, would be expected to be minimal.

Another limitation of this study is the small validation size of only 10 patients. Only 9 patient datasets that had been previously contoured with multiple metastases within the department and not contained within the model could be used for validation. The TROG data set was used as the 10th validation case. Although the 10 datasets had varying locations and sizes on PTV’s contoured, a larger validation set would have created a stronger comparison case.

## Conclusion

When treating multiple intracranial metastases, there was increased conformity of the prescription dose to the PTV using a si‐VMAT technique compared to our current mi‐SRS technique utilising cones or dynamic arcs. Although the volume of brain receiving between 15 Gy and 7.5 Gy was greater for the si‐VMAT technique, the extra volume treated is a very small percentage of the overall brain volume. In assessing the 3 Gy dose to the brain, the mi‐ SRS approach was superior. In relation to the utilisation of current resources and time savings, the si‐VMAT technique presents the greatest advantages for both planning and treatment, with time savings in both areas increasing with the number of metastases being treated.

## Conflict of Interest

The authors declare no conflict of interest.
